# Cigarette smoke exposure reduces hemorrhagic shock induced circulatory dysfunction in mice with attenuated glucocorticoid receptor function

**DOI:** 10.3389/fimmu.2022.980707

**Published:** 2022-09-12

**Authors:** Martin Wepler, Jonathan M. Preuss, Cornelia Tilp, Martina Keck, Jochen Blender, Ulrich Wachter, Tamara Merz, Josef Vogt, Sandra Kress, Michael Gröger, Andrea Hoffmann, Marina Fink, Enrico Calzia, Ute Burret, Peter Radermacher, Jan P. Tuckermann, Sabine Vettorazzi

**Affiliations:** ^1^ Department of Anesthesiology and Intensive Care Medicine, University Hospital, Ulm, Germany; ^2^ Institute for Anesthesiologic Pathophysiology and Process Engineering, Ulm University, Ulm, Germany; ^3^ Institute of Comparative Molecular Endocrinology (CME), Ulm University, Ulm, Germany; ^4^ Immunology and Respiratory, Boehringer Ingelheim Pharma GmbH & Co KG, Biberach, Germany

**Keywords:** catecholamines, cigarettes smoke exposure, glucocorticoid receptor function, hemodynamic function, hemorrhagic shock, metabolic function, inflammation

## Abstract

**Introduction:**

We previously showed that attenuated glucocorticoid receptor (GR) function in mice (GR^dim/dim^) aggravates systemic hypotension and impairs organ function during endotoxic shock. Hemorrhagic shock (HS) causes impaired organ perfusion, which leads to tissue hypoxia and inflammation with risk of organ failure. Lung co-morbidities like chronic obstructive pulmonary disease (COPD) can aggravate tissue hypoxia *via* alveolar hypoxia. The most common cause for COPD is cigarette smoke (CS) exposure. Therefore, we hypothesized that affecting GR function in mice (GR^dim/dim^) and pre-traumatic CS exposure would further impair hemodynamic stability and organ function after HS.

**Methods:**

After 3 weeks of CS exposure, anesthetized and mechanically ventilated GR^dim/dim^ and GR^+/+^ mice underwent pressure-controlled HS for 1h *via* blood withdrawal (mean arterial pressure (MAP) 35mmHg), followed by 4h of resuscitation with re-transfusion of shed blood, colloid fluid infusion and, if necessary, continuous intravenous norepinephrine. Acid–base status and organ function were assessed together with metabolic pathways. Blood and organs were collected at the end of the experiment for analysis of cytokines, corticosterone level, and mitochondrial respiratory capacity. Data is presented as median and interquartile range.

**Results:**

Nor CS exposure neither attenuated GR function affected survival. Non-CS GR^dim/dim^ mice had a higher need of norepinephrine to keep target hemodynamics compared to GR^+/+^ mice. In contrast, after CS exposure norepinephrine need did not differ significantly between GR^dim/dim^ and GR^+/+^ mice. Non-CS GR^dim/dim^ mice presented with a lower pH and increased blood lactate levels compared to GR^+/+^ mice, but not CS exposed mice. Also, higher plasma concentrations of some pro-inflammatory cytokines were observed in non-CS GR^dim/dim^ compared to GR^+/+^ mice, but not in the CS group. With regards to metabolic measurements, CS exposure led to an increased lipolysis in GR^dim/dim^ compared to GR^+/+^ mice, but not in non-CS exposed animals.

**Conclusion:**

Whether less metabolic acidosis or increased lipolysis is the reason or the consequence for the trend towards lower catecholamine need in CS exposed GR^dim/dim^ mice warrants further investigation.

## Introduction

Hemorrhagic shock (HS) is one of the major mediators of trauma-related mortality (1). During HS as well as during resuscitation from HS impaired organ blood flow may lead to tissue hypoxia, resulting in risk of organ damage (2). Tissue hypoxia itself induces inflammation (3), which in turn aggravates the risk of organ damage. Both, tissue hypoxia and inflammation may lead to enhanced radical formation and mitochondrial dysfunction, altogether leading to multiorgan failure (MOF (1, 2, 4),). During resuscitation after HS, it is important to maintain mean arterial pressure (MAP) to ensure perfusion of the most important organs as heart, kidney, liver, lung, or brain. At best, this hemodynamic state enables microcirculatory perfusion of these important organs to prevent longer impairment of O_2_-availability and, thus irreversible organ dysfunction or damage (5). On a cellular level, impaired O_2_-availability may lead to mitochondrial dysfunction with uncoupling of mitochondrial respiration and a lack of mitochondrial substrates, resulting in reduced production of adenosine triphosphate (ATP) and increased oxidative stress (5). Besides this mitochondrial dysfunction due to a reduced O_2_-availability, during and after shock a hypermetabolic condition with insulin resistance and increased oxygen demands often occurs (6). This, in turn, causes impaired glucose and oxygen availability on a cellular level and, ultimately, lead to metabolic failure. Together with the mitochondrial dysfunction this metabolic failure further impairs organ function after shock (7).

Our group showed already that pre-traumatic cigarette-smoke (CS) exposure enhanced post-traumatic inflammation and radical stress, thereby aggravating lung dysfunction (8). CS is the major risk factor for chronic obstructive pulmonary disease (COPD) (9) and COPD as a lung co-morbidity increases pulmonary and systemic inflammation and might increase tissue hypoxia *via* alveolar hypoxia (10). Furthermore, CS exposure-induced COPD aggravated acute lung injury (ALI) in a murine model of blunt chest trauma (8, 11), causing the strongest inflammatory response in mice after HS and resuscitation under intensive care treatment (12).

Glucocorticoids (GCs) mediate their effects through the glucocorticoid receptor (GR) (13). The GR is an intracellular, ligand-activated transcription factor, which regulates gene transcription by several mechanisms: as a protein dimer it can bind palindromic DNA sequences (glucocorticoid response elements – GREs) or DNA half sites as a monomeric protein (GR monomer (14)). In a non-resuscitated mouse model of endotoxemia, we previously showed that the GR is crucial for the resolution of systemic inflammation (15). In a more recent study, we demonstrated that intact GR dimer also mediates hemodynamic stability in a resuscitated mouse model of endotoxemia, indicated by a higher need of catecholamines in mice with a impaired GR dimerization function (GR^dim/dim^) to keep hemodynamic targets (16). In addition to the mouse model of endotoxemia, we already showed that HS led to impaired lung mechanics and aggravated lung inflammation in GR^dim/dim^ mice compared to littermate control wildtype mice (GR^+/+^) (17). This previous study showed that functional GR signaling plays a crucial role to attenuate HS-induced lung damage. Based on this study, we hypothesized that an attenuation of GR function together with CS exposure as a lung co-morbidity may lead to organ dysfunctions due to metabolic disturbances and an increased hemodynamic instability during resuscitation from HS with the effects on lung function already published (17). To ensure clinical transferability of the results, we used our murine model of HS with resuscitation similar to intensive care management (infusion of crystalloids and norepinephrine (NE) to achieve hemodynamic targets, lung-protective mechanical ventilation, measurements of gas exchange, and control of body temperature (17–19)) together with a detailed analysis of the metabolic state (18, 20) and mitochondrial function (16, 21) in CS exposed animals (21).

## Material and methods

This study was approved by the federal authorities for animal research of the Regierungspräsidium Tübingen, Baden-Wuerttemberg, Germany (approved animal experimentation number: 1359, August 17^th^, 2017), and performed in adherence with the National Institutes of Health Guidelines on the Use of Laboratory Animals and the European Union ‘‘Directive 2010/63 EU on the protection of animals used for scientific purposes.’’ GR^dim/dim^ mice (Nr3c1tm3Gsc) (22) were bred in a mixed background (129/SvEv x C57BL/6) and housed in the animal facility at University Ulm or in the housing facility at Boehringer Ingelheim (Biberach). GR^+/+^ littermate controls were used as wild-type mice. Animals always had free access to food and water, were kept under standardized conditions, and were equally distributed in terms of age (16-26 weeks) and body weight (23-34 g). Groups comprised 7 animals (GR^+/+^ non-CS), 9 animals (GR^dim/dim^ non-CS), 8 animals (GR^+/+^ CS exposure), and 9 animals (GR^dim/dim^ CS exposure) of both gender. Due to the complicated surgery 2 animals in the GR^+/+^ non-CS group and 1 animal in the GR^+/+^ CS group deceased during surgery.

### Implementation of general anesthesia and surgery

Anesthesia was induced *via* sevoflurane (2.5%; sevoflurane, Abbott, Wiesbaden, HE, Germany) as described previously (19, 21), followed by intraperitoneal injection (ip) of ketamine (120µg·g^-1^; Ketanest-S, Pfizer, New York City, NY), midazolam (1.25µg·g^-1^; Midazolam-ratiopharm, Ratiopharm, Ulm, BW, Germany) and fentanyl (0.25µg·g^-1^; Fentanyl-hameln, Hameln Pharma Plus GmbH, Hameln, NI, Germany). Afterwards, animals were placed on a closed-loop-system for body temperature control (19, 21). Lung-protective mechanical ventilation using a small animal ventilator (FlexiVent, Scireq, MO, Canada) was performed *via* a tracheostomy (19, 21). Surgical instrumentation comprised catheters in the jugular vein for fluid administration, the carotid artery for hemodynamic measurements, the femoral artery for blood removal, and the bladder to collect urine (19, 21). General anesthesia was titrated to guarantee complete tolerance against noxious stimuli and was sustained by continuous intravenous (iv) administration of ketamine, midazolam, and fentanyl to reach deep sedation (0.85 [0.79; 0.88] μl·g^-1^·min.^-1^ for all animals). Animals were mechanically ventilated with ventilator settings being F_i_O_2_ 0.21%, respiratory rate 150·min^-1^, tidal volume of 6 mL·kg^-1^, and inspiratory/expiratory time ratio 1:2. Ventilation was modified to maintain an arterial PaCO_2_ between 30 mmHg and 40 mmHg throughout the experiment as described previously (21). Therefore, blood gas analyses were performed before HS, 2h after re-transfusion of shed blood, and at the end of the experiment. However, P_a_CO_2_ values reported in **Table 1** were measured at the end of experiment where adaptation of ventilation was no longer practicable. Therefore, P_a_CO_2_ values may vary and appear out of that range at the end of the experiment. Positive end-expiratory pressure (PEEP) was adjusted according to the arterial PaO_2_ (PaO_2_/F_i_O_2_-ratio>300mmHg: PEEP=3cmH_2_O; PaO_2_/FiO_2_-ratio<300mmHg: PEEP=5cmH_2_O; PaO_2_/FiO_2_-ratio<200mmHg: PEEP=8cmH_2_O) (19, 21). Recruitment maneuvers (5s hold at 18 cmH_2_O) were repeated hourly to avoid any impairment of thoraco-pulmonary compliance due to anesthesia- and/or supine position-induced atelectasis. A detailed description of the intensive care measures in mice performed in the present study (mouse intensive care unit, MICU) can be found elsewhere (23).

**Table 1:** Hemodynamic and metabolic measurements as well as ventilation parameters in GR^dim/dim^ and GR^+/+^ mice with or without cigarette-smoke (CS) exposure and after hemorrhagic shock(HS) with subsequent resuscitation (4h) at the end of the experiment.

Parameters	Resuscitation (IV colloids and norepinephrine)			
	GR^+/+^+non-CS (n = 7)	GR^dim/dim^+non-CS (n = 9)	GR^+/+^+CS (n = 8)	GR^dim/dim^+CS (n = 9)
Bodyweight [g]	27.5 (25.6; 27.8)	26.7 (25.2; 30.6)	29.2 (26.4; 32.4)	27.3 (25.6; 30.1)
Heart Rate [beats·min^-1^]	372 (352; 439)	480 (413; 538)	307 (276; 506)	**580 (553; 593)^§^**
Mean Arterial Pressure [mmHg]	66 (56; 77)	54 (50; 64)	57 (53; 63)	62 (55; 72)
Horovitz Index [mmHg]	486 (386; 490)	356 (348; 457)	460 (358; 525)	322 (293; 384)
PaCO_2_ [mmHg]	29 (28; 38)	38 (32; 43)	35 (29; 37)	**43 (41; 46)^§^**
Minute ventilation [mL·kg^-1^·min^-1^]	1100 (1040; 1150)	1100 (980; 1150)	1000 (943; 1150)	1070 (1050; 1110)
Glucose [mg·dL^-1^]	115 (106; 123)	120 (110; 162)	96 (87; 107)	**128 (122; 134)^§^**
Arterial pH	7.38 (7.33; 7.44)	7.23 (7.10; 7.31)*	7.35 (7.23; 7.42)	7.26 (7.23; 7.32)
Arterial base excess [mmol·L^-1^]	-6.4 (-7.4; -5.4)	-10.2 (-15.7; -8.0)*	-7.1 (-10.4; -5.6)	**-7.6 (-8.9; -4.8)^$^**
Lactate [mmol·L^-1^]	1.2 (0.8; 1.5)	2.3 (1.4; 4.6)*	1.3 (1.1; 1.6)	**1.2 (1.1; 1.7)^$^**
Hemoglobin [g·dL^-1^]	8.0 (7.5; 8.4)	7.1 (6.7; 7.6)	8.7 (7.7; 9.2)	**8.7 (7.8; 9.5)^$^**
Blood volume withdrawn for shock [μl·g^-1^]	30.0 (30.0; 30.0)	30.0 (27.5; 30.0)	30.0 (30.0; 30.0)	30.0 (23.0; 30.0)
Urinary output [µL]	2235 (1486; 3132)	2118 (1542; 3191)	1760 (1303; 4130)	1945 (1083; 2444)

*P < 0.05 vs. GR^+/+^ non-CS. ^§^P < 0.05 vs. GR^+/+^ CS. ^$^P < 0.05 vs. GR^dim/dim^ non-CS. Data is shown as median (25^th^ and 75^th^ percentile). Significant different values are highlighted bold.

### Cigarette smoke inhalation procedure

CS exposure was performed for 5 days per week over a period of 3 weeks using a standardized protocol as described previously (8, 11, 24). Particle concentration was monitored by a real time ambient particle monitor (MicroDust Pro, Casella, Amherst, NH, USA). In pilot experiments, this CS-exposure procedure had not caused any effect on the behavior, body weight, or respiratory pattern. Control animals (non-CS) were exposed to room air. After CS exposure, mice were transported from Boehringer Ingelheim (Biberach) to Ulm University and there were allowed to recover for 1 week to avoid acute stress effects induced by the CS procedure per se (11, 25).

### Induction of hemorrhagic shock

After the initiation of lung-protective mechanical ventilation and instrumentation for vascular access, mice underwent 1h of HS. HS was performed by removing blood out of the femoral catheter with citrate-coated syringes (about 30µL·g^-1^) to titrate mean arterial pressure (MAP) to 35 mmHg. Blood was further removed or re-transfused to keep MAP at 35 mmHg for 1h. Besides, fluid administration was temporarily stopped. At the start of the resuscitation phase, shed blood was re-transfused, together with the administration of hydroxyethyl starch 6% (10μl·g^-1^ h^-1^) and NE dissolved in balanced electrolyte solution (≤0.5 ml·h^-1^ for all animals) *via* the jugular vein catheter titrated to maintain MAP≥55 mmHg. The bladder was punctured to collect urine during the experiment. After 4h of resuscitation, animals were exsanguinated, blood and tissue samples were taken immediately thereafter, and prepared for further analyses (19, 21).

### Analysis of metabolic pathways and kidney function

Thirty minutes before induction of hemorrhagic shock, mice received a primed continuous infusion of stable, non-radioactive-labeled ^13^C_6_-glucose, ^15^N_2_-urea, ^2^H_5_-glycerol, and 5,5,5–^2^H_3_-leucine. Expiratory breath gas was sampled 0.5, 1.5, 2.5, 3.5, 4.5, and 5.5 h (end of experiment) after start of the stable isotope infusion. Arterial blood was sampled at time points 0.5, 2.5, 4.5, and 5.5 h. Together with urine output, gas chromatography/mass spectrometry measurement of plasma und urinary creatinine concentrations using ^2^H_3_-creatinine as internal standard allowed for calculating creatinine clearance (18). Expiratory gas was analyzed immediately after collection by gas chromatography/mass spectrometry (GC/MS) for total expiratory CO_2_ concentration and ^13^CO_2_ enrichment to assess glucose oxidation rate. Plasma and urine samples were stored at −80°C until sample workup. For metabolic analysis, we only analyzed animals that survived the whole observation period (4 h) in order to guarantee a steady state of isotopes (n=7 for GR^dim/dim^ and GR^+/+^ non-CS, n=8 for GR^+/+^ CS, and n=9 for GR^dim/dim^ CS). For further details see **supplemental material**.

### Parameters of hemodynamics, gas exchange, and metabolism

Systemic hemodynamics and body temperature were recorded hourly. Blood gas tensions, acid-base status, glycaemia, and lactatemia were assessed at the end of the resuscitation period *via* arterial blood gas analysis (ABL800 Felx; Radiometer, Krefeld, Germany) (19, 21).

### Mitochondrial respiration

Mitochondrial respiratory capacity was determined *via* high-resolution respirometry with a clark-electrode-based system (Oxygraph 2k, OROBOROS Instruments Corp., Innsbruck, Austria) as described previously (19). Post-mortem muscle, heart, liver, and brain biopsies were mechanically homogenized in Mir05 (respiration medium). Mir05 is composed of 0.5mM EGTA, 3mM MgCl2·6H2O, 60mM Lactobionic acid, 20mM Taurine, 10mM KH2PO4, 20mM HEPES, 110mM Sucrose, 1g·L-1 bovine serum albumin). 1.5-2 mg of tissue (1.5mg: heart, 2mg tissue: muscle, liver, and brain) were added to the Oxygraph chamber. By the addition of a defined sequence of substrates and inhibitors, various states of mitochondrial function could be assessed. Complex I activity was determined after addition of 10mM pyruvate, 10mM glutamate, 5mM malate and 5mM ADP. 10µM cytochrome c was added to check for mitochondrial integrity. Maximum oxidative phosphorylation (OxPhos) was evaluated after subsequent addition of 1mM octanoyl-carnitine and 10mM succinate. Leak compensation was assessed after inhibition of the ATP-synthase by 2.5µM oligomycin, followed by stepwise titration of the uncoupling agent Carbonyl cyanide-4-(trifluoromethoxy)-phenylhydrazone (FCCP, final concentration 1.5µM) to reach maximum respiratory activity of the electron transfer system in the uncoupled state (ETS).

### Measurements of cytokine, chemokine, and corticosterone concentrations in plasma

Cytokines, chemokines, and growth factors were measured using Bio-Plex Pro Mouse Cytokine 23-plex Assay (Group I) (Biorad) simultaneously in the plasma according to the manufacturer´s protocol. The multiplex-assay was performed with Bio-Plex 200 machine (Biorad) and the Bio-Plex Manager TM 6.1 software (Biorad). Plasma corticosterone levels were determined with an enzyme linked immunosorbent assay (ELISA) kit (TECAN, IBL international GmbH, RE52211) according to the manufactures protocol. In short, plasma samples were incubated in the anti-corticosterone antibody coated wells alongside with a horse-reddish peroxide (HRP) conjugated corticosterone competitor for one hour at room temperature. After five subsequent washing steps, HRP substrate was added for 15 min followed by stop solution and immediate measure of color intensity at 450 nm using Fluostar OPTIMA plate reader. Corticosterone concentrations were calculated according to the provided standards using a 4-parametric fit.

### Statistical analysis

Unless stated otherwise, all data are presented as median (25^th^ and 75^th^ percentile). Data sets were analyzed using non-parametric statistics, i.e. Mann-Whitney U-test (one factor, two independent samples) or Kruskal-Wallis test with *post hoc* Dunn’s comparison testing (one factor, four independent samples). P-values <0.05 were considered statistically significant. Quantitative graphical presentations and statistical analyses were accomplished by using GraphPad Prism 9 (GraphPad Software Inc, La Jolla, Calif).

## Results

### Survival

All CS exposed animals survived to the end of the observation period during resuscitation from GR^dim/dim^ HS (4h). Only two animals with an attenuation of GR function and non-CS exposure did not reach the end of the observation period, however, the survival rates did not show any significant intergroup differences and have been reported previously in the **supplementary materials** of a publication of our group (17).

### Hemodynamics, acid–base balance, parameters of organ (dys)function as well as mitochondrial respiration

**Figure 1:**
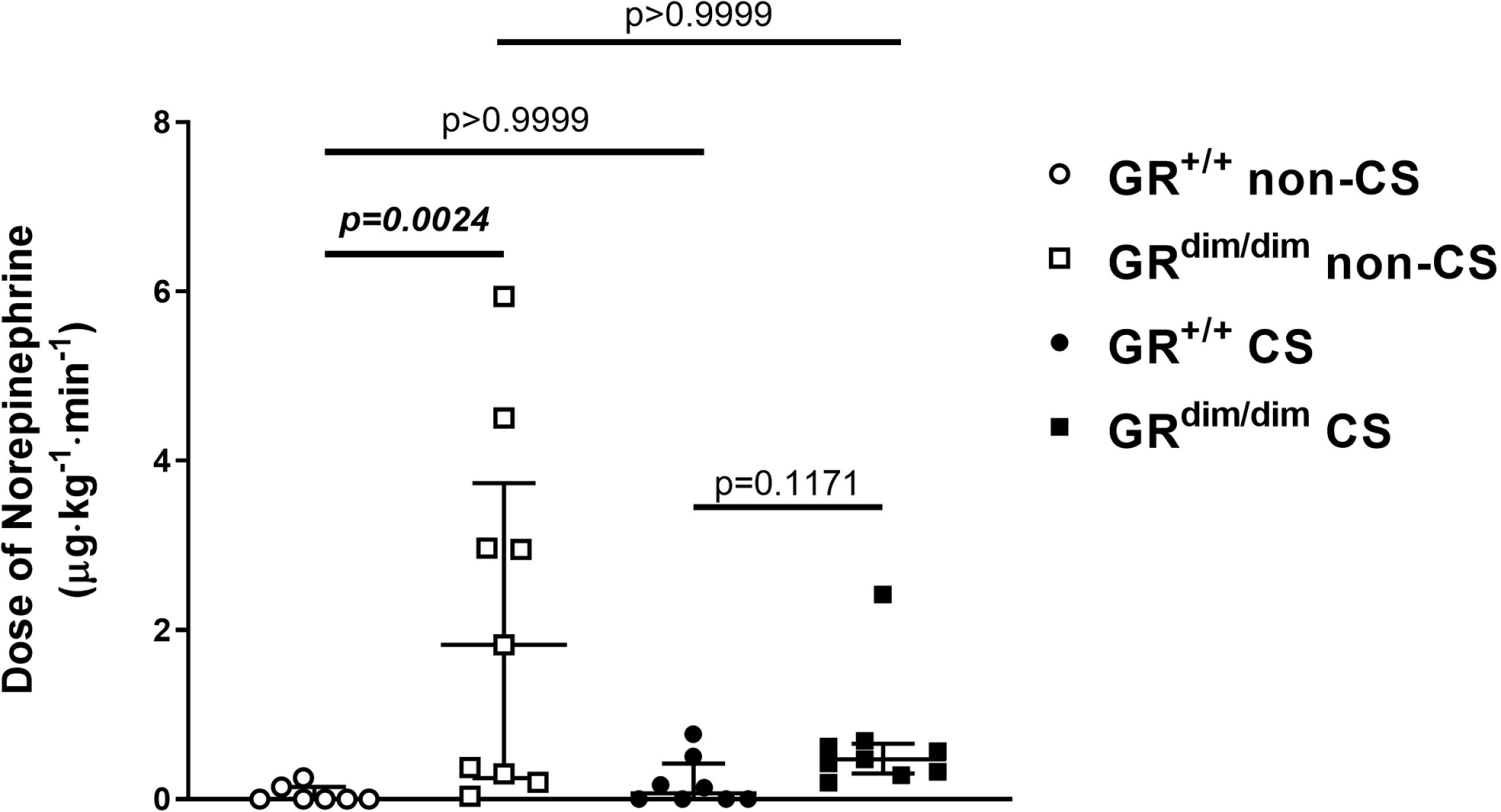
Doses of norepinephrine(NE) in mechanically ventilated GR^dim/dim^ and GR^+/+^ mice with or without cigarette-smoke (CS) exposure and after 1 h of hemorrhagic shock (HS, mean arterial pressure (MAP) 35 mmHg) and subsequent resuscitation (colloids, NE) for 4 h. NE was titrated intravenously during resuscitation to keep MAP ≥55mmHg. GR^+/+^ mice non-CS: n = 7, GR^dim/dim^ mice non-CS: n = 9, GR^+/+^ mice CS: n=8, GR^dim/dim^ mice CS: n = 9. Data is presented as median (25^th^ and 75^th^ percentile and minimum/maximum).

**Figure 2:**
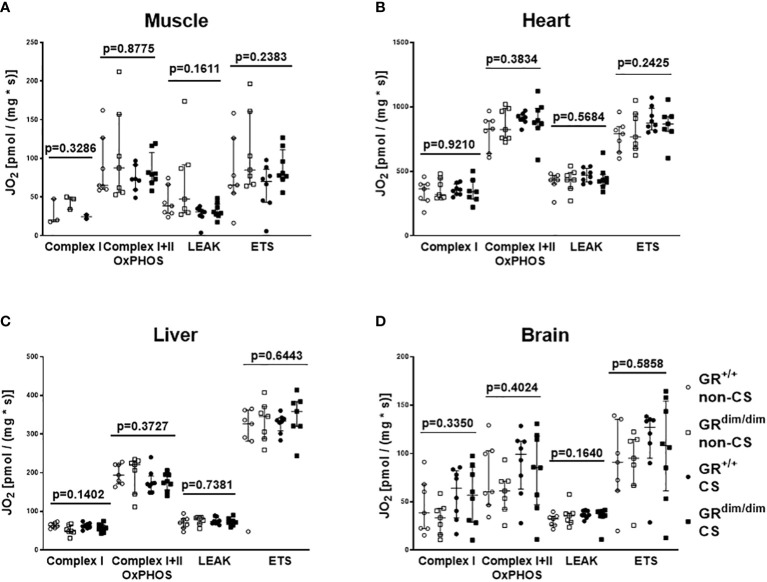
Mitochondrial respiration (JO2) in tissue from muscle **(A)**, heart **(B)**, liver **(C)**, and brain **(D)** in GR^dim/dim^ and GR^+/+^ mice with or without cigarette-smoke (CS) exposure and after 1 h of hemorrhagic shock (HS, MAP 35 mmHg) and subsequent resuscitation (colloids, NE) for 4 h. Overall p-values from Kruskal-Wallis-Test are shown above. GR^+/+^ mice non-CS: n = 3-7, GR^dim/dim^ mice non-CS: n = 3-7, GR^+/+^ mice CS: n = 2-8, GR^dim/dim^ mice CS: n=8. For the group GR^dim/dim^ CS in muscle tissue there were no measurements available due to a measurement problem. Data is presented as median (25^th^ and 75^th^ percentile and minimum/maximum).

To examine the effects of an attenuated GR function as well as exposure of CS on organ function during resuscitation from 1h of HS, hemodynamics, metabolic parameters, and mitochondrial respiration were investigated. **Table 1** summarizes the parameters of hemodynamics and acid–base status. Heart rate was significantly higher in GR^dim/dim^ compared to GR^+/+^ mice with CS-exposure, whereas mean arterial pressure (MAP) did not differ between groups. In non-CS exposed mice, GR^dim/dim^ mice had a significantly higher need of catecholamines to keep hemodynamic stability compared to GR^+/+^ mice (**Figure 1**). In contrast, need of catecholamines did not differ in CS-exposed GR^dim/dim^ and GR^+/+^ mice (**Figure 1**). After CS exposure, arterial partial pressure of carbon dioxide (P_a_CO_2_) was significantly higher in GR^dim/dim^ compared to GR^+/+^ mice, whereas minute ventilation did not differ between groups (**Table 1**). In non-CS exposed mice, pH as well as base excess (BE) were lower and lactate levels were higher in GR^dim/dim^ compared to GR^+/+^ mice (**Table 1**). Because increased lactate levels may be linked to disturbances in mitochondrial respiration (6, 26), important metabolic organs as muscle, heart, liver and brain were investigated for mitochondrial respiration in the current study. However, mitochondrial respiration (**Figure 2**) in tissue from muscle, heart, liver, and brain in GR^dim/dim^ and GR^+/+^ mice with or without CS exposure revealed no intergroup differences in all four organs analyzed in the current study. In summary, after resuscitation from HS, the attenuated function of the GR resulted in a higher need of catecholamines to keep hemodynamic stability and a lower pH together with increased lactate levels. These differences were not present in CS exposed mice.

### Measurements of plasma cytokine levels at the end of 4 hours of resuscitation after hemorrhagic shock

To determine effects of an attenuation of GR function and CS exposure on inflammatory parameters, plasma concentration of cytokines and chemokines were determined. In the non-CS group, concentration of interleukine-6 (IL-6), interleukine-12 p40 (IL-12 p40), and Eotaxin were significantly higher in plasma of GR^dim/dim^ mice compared to GR^+/+^ mice (**Table 2**). Interestingly, there were no intergroup differences for the measured plasma cytokines when animals were exposed to CS before HS. Therefore, only the attenuation of GR function led to an increase in some plasma cytokines, whereas this increase was prevented by CS pre-exposure.

**Table 2:** Concentration of cytokines in plasma in GR^dim/dim^ and GR^+/+^ mice with or without cigarette-smoke (CS) exposure and after HS with subsequent resuscitation (4 h) at the end of the experiment.

Concentration of cytokines in plasma	Resuscitation (IV colloids and NE)			
	GR^+/+^+non-CS(n = 1-6)	GR^dim/dim^+non-CS(n = 3-9)	GR^+/+^+CS(n = 1-8)	GR^dim/dim^+CS(n = 1-9)
Interleukine-1 alpha (IL-1 α) [pg·ml^-1^]	20 (18; 44)	18 (13; 28)	18 (15; 22)	20 (14; 23)
Interleukine-1 beta (IL-1 β) [pg·ml^-1^]	1.8 (0.6; 6.2)	10.9 (4.1; 15.6)	3.0 (3.0; 5.2)	3.0 (0.6; 7.4)
Interleukine-2 (IL-2) [pg·ml^-1^]	6.3 (4.7; 7.8)	5.3 (3.4; 13.0)	1.7 (1.7; 1.7)	3.4 (3.4; 3.4)
Interleukine-3 (IL-3) [pg·ml^-1^]	0.5 (0.1; 1.2)	2.4 (1.0; 2.9)	1.0 (0.3; 7.7)	1.2 (0.5; 2.4)
Interleukine-4 (IL-4) [pg·ml^-1^]	5.1 (2.9; 7.0)	3.4 (2.3; 4.8)	4.3 (2.0; 5.3)	2.5 (1.4; 3.6)
Interleukine-5 (IL-5) [pg·ml^-1^]	10.0 (7; 23)	34 (20; 37)	13 (8; 26)	22 (17; 39)
Interleukine-6 (IL-6) [pg·ml^-1^]	170 (69; 285)	451 (330; 566)*	297 (205; 393)	327 (212; 405)
Interleukine-9 (IL-9) [pg·ml^-1^]	11 (9; 13)	6 (5; 8)	25 (3; 41)	86 (66; 106)
Interleukine-10 (IL-10) [pg·ml^-1^]	10 (10; 22)	22 (13; 47)	16 (8; 20)	9 (8; 23)
Interleukine-12 p40 (IL-12 p40) [pg·ml^-1^]	142 (103; 191)	273 (218; 518)*	192 (148; 224)	200 (140; 221)
Interleukine-12 p70 (IL-12 p70) [pg·ml^-1^]	4 (4; 4)	75 (24; 176)	42 (36; 48)	45 (39; 50)
Interleukine-13 (IL-13) [pg·ml^-1^]	25 (17; 40)	23 (15; 30)	17 (11; 48)	15 (12; 29)
Interleukine-17 (IL-17) [pg·ml^-1^]	4.5 (2.5; 7.2)	4.9 (2.5; 15.4)	5.1 (4.1; 5.7)	3.7 (1.0; 4.9)
Eotaxin [pg·ml^-1^]	1100 (810; 1802)	2729 (1582; 3275)*	1820 (1563; 2101)	2536 (1782; 2824)
Granulocyte Colony-stimulating Factor (G-CSF) [pg·ml^-1^]	1042 (704; 1277)	1333 (960; 2310)	1310 (1247; 1344)	879 (830; 1395)
Granulocyte-macrophage Colony-stimulating Factor (GM-CSF) [pg·ml^-1^]	27 (27; 34)	37 (18; 56)	37 (15; 64)	22 (17; 27)
Interferon gamma (INF-γ) [pg·ml^-1^]	2.5 (1.6; 3.9)	3.8 (2.9; 6.4)	3.3 (2.9; 10.8)	4.7 (2.9; 7.8)
Keratinocyte Chemoattractant (KC) [pg·ml^-1^]	20 (17; 28)	18 (13; 22)	25 (19; 34)	28 (19; 57)
Monocyte Chemoattractant Protein-1 (MCP-1) [pg·ml^-1^]	124 (37; 147)	258 (199; 751)	129 (78; 173)	184 (90; 299)
Macrophage Inflammatory Protein-1 alpha (MIP-1 α) [pg·ml^-1^]	0.7 (0.3; 0.9)	1.0 (0.7; 1.9)	0.8 (0.7; 1.3)	0.6 (0.3; 0.8)
Macrophage Inflammatoryt Protein-1 beta (MIP-1 β) [pg·ml^-1^]	5.0 (5.0; 5.0)	7.0 (2.7; 10.8)	5.0 (5.0; 5.0)	46.1 (0.0; 92.1)
Regulated And Normal T-cell Expressed and Secreted (RANTES) [pg·ml^-1^]	6.2 (5.2; 14.7)	34.6 (10.8; 138.1)	8.0 (5.5; 12.2)	8.3 (6.2; 12.3)
Tumor Necrosis Factor alpha (TNF-α) [pg·ml^-1^]	21 (14; 47)	34 (11; 38)	15 (9; 26)	14 (14; 14)

*P < 0.05 vs. GR^+/+^ non-CS. Data is shown as median (25^th^ and 75^th^ percentile). Signifiant different values are highlighted bold.

### Effects of an attenuation of GR function and CS exposure on metabolic parameters

**Figure 3:**
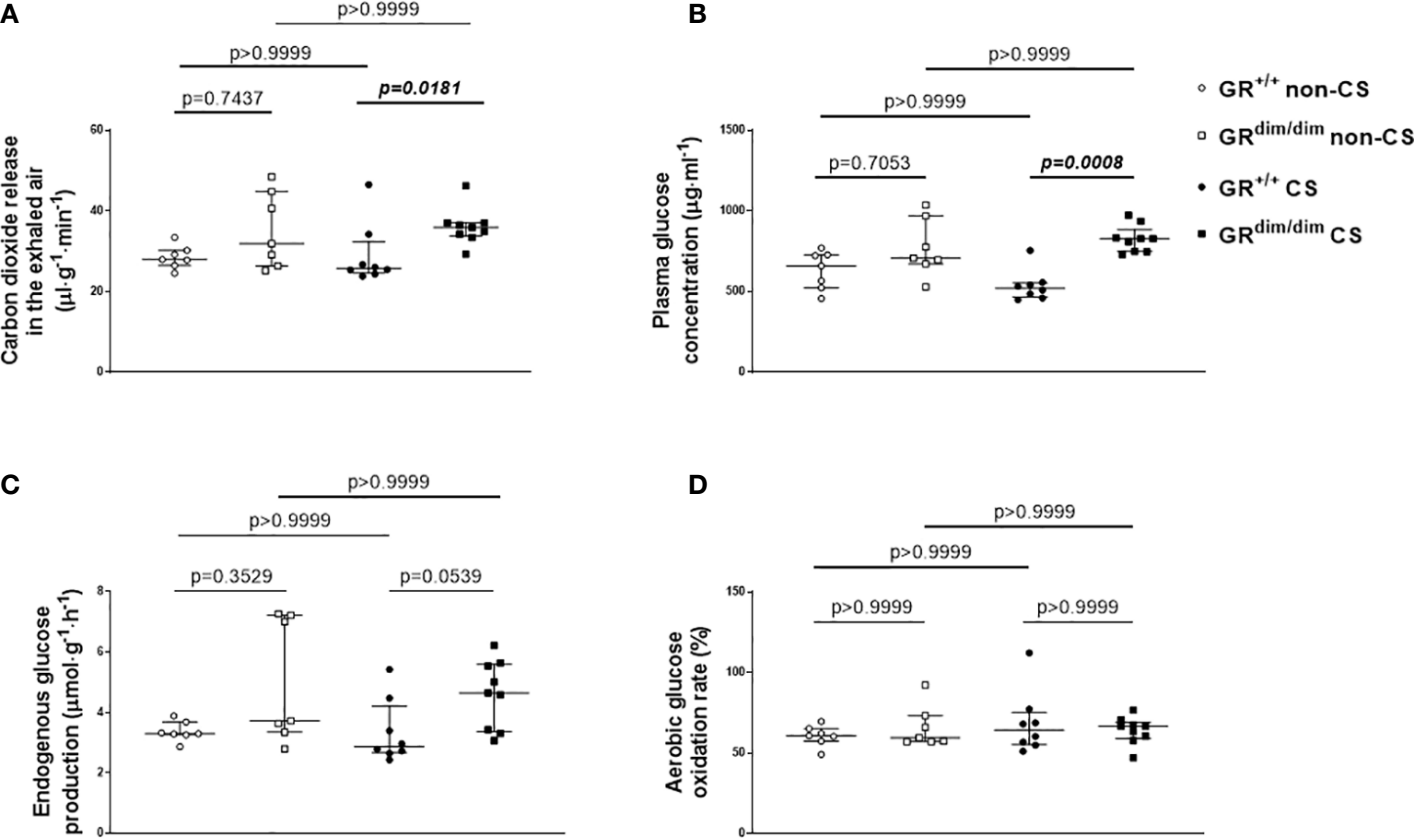
Glucose metabolism. **(A)** Carbon dioxide release *via* the exhaled air, **(B)** plasma glucose concentration, **(C)** endogenous glucose production rate, and **(D)** glucose oxidation as percentage of oxidation of infused tracer. GR^+/+^ mice non-CS: n=7, GR^dim/dim^ mice non-CS: n = 7, GR^+/+^ mice CS: n = 8, GR^dim/dim^ mice CS: n = 9. Data is presented as median (25^th^ and 75^th^ percentile and minimum/maximum).

**Figure 4:**
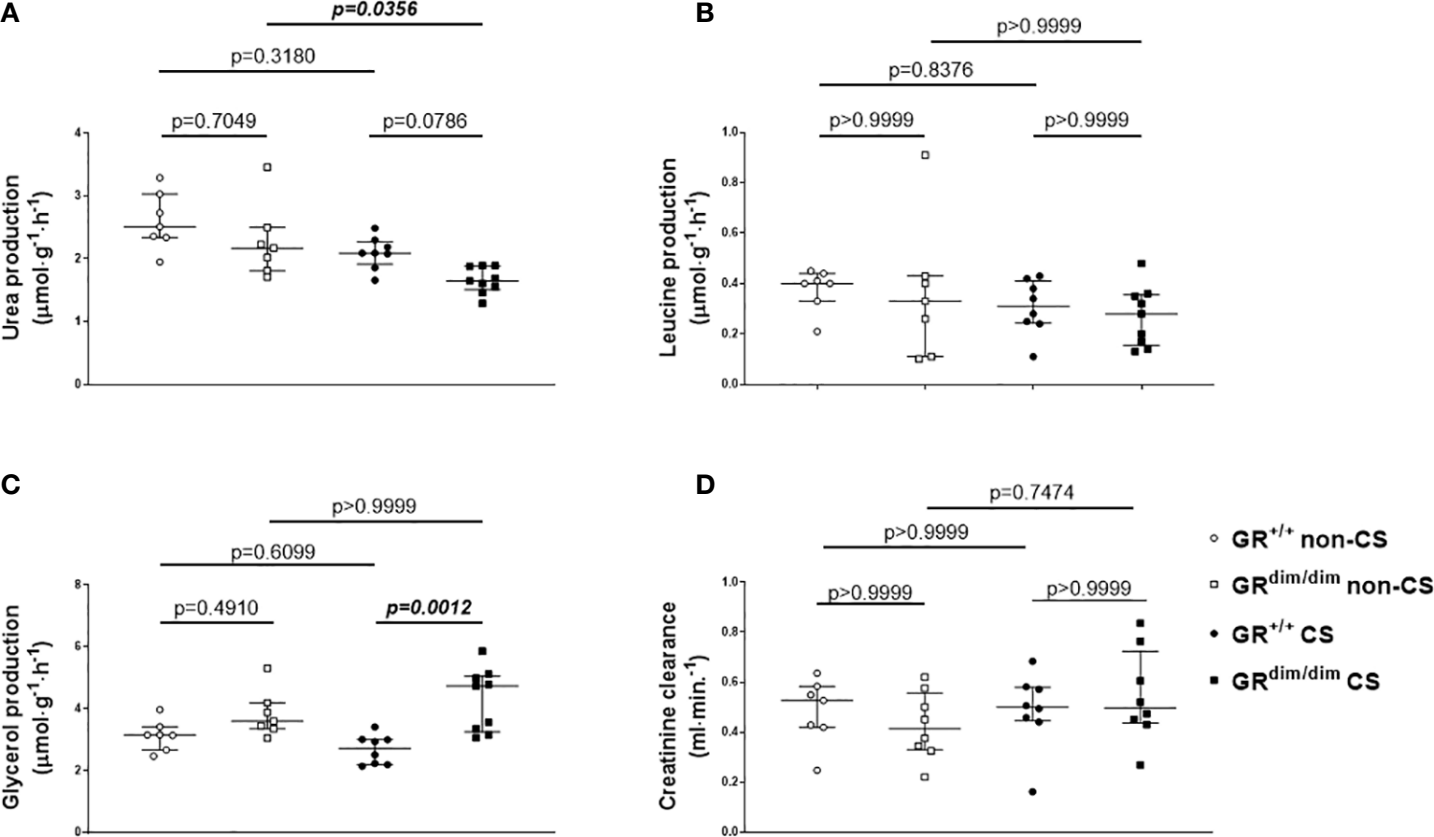
Parameters of metabolic and kidney function. Rates of appearance of **(A)** urea, **(B)** leucine, **(C)** glycerol as well as **(D)** creatinine clearance, as measures for hepatic metabolic capacity, protein degradation, lipolysis, and kidney function, respectively. ^*^P < 0.05. ^#^P < 0.01. GR^+/+^ mice non-CS: n = 7, GR^dim/dim^ mice non-CS: n = 7-8, GR^+/+^ mice CS: n=8, GR^dim/dim^ mice CS: n = 8-9. Data is presented as median (25^th^ and 75^th^ percentile and minimum/maximum).

Despite no differences in minute ventilation during resuscitation (**Table 1**), GR^dim/dim^ mice showed higher CO_2_ values in the exhaled air compared to GR^+/+^ mice, both after CS exposure during the observation period (**Figure 3A**, together with increased arterial partial pressure of CO_2_, see **Table 1**). The same genotype dependent difference appeared for the concentration of glucose in the plasma, whereas the rate of endogenous glucose production as well as glucose oxidation rate showed no inter-group differences (**Figures 3C, D**). After CS exposure, rate of appearance for urea (marker for hepatic metabolic capacity as well as kidney function) was significantly lower in GR^dim/dim^ mice compared to GR^dim/dim^ mice with no CS exposure (**Figure 4A**). Measures of lipolysis (measured *via* rate of appearance for glycerol), protein degradation (rate of appearance for leucine), and kidney function (creatinine clearance, urine output [**Table 1**]) showed no intergroup differences (**Figures 4C, D**). Taken together, during resuscitation after HS, CS exposed GR^dim/dim^ mice presented with an increased glucose concentration in plasma (**Figure 3B**), which does not come from an increase in gluconeogenesis or decrease in glucose oxidation (**Figures 3C, D**). Furthermore, due to no differences in kidney function (creatinine clearance, **Figure 4B**) as well as protein breakdown (rate of appearance for leucine, **Figure 4D**), the reduced rate of appearance of urea in CS exposed GR^dim/dim^ mice (**Figure 4A**) is most likely a result of a reduced hepatic synthesis.

### Measurements of corticosterone concentration in plasma

As the above measured parameters are well known to be influenced by corticosteroids, we determined the concentration of corticosterone in plasma. Neither an attenuation of GR function nor CS exposure had any effects on plasma corticosterone levels in mice in the present study under intensive care conditions (non-CS GR^+/+^: 112 (78;141), non-CS GR^dim/dim^: 85 (35; 156); CS GR^+/+^: 93 (65; 147), CS GR^dim/dim^: 82 (55; 119), P=0.6852).

## Discussion

In the present study, we examined the effects of a dysfunction in the glucocorticoid receptor (GR^dim/dim^) on hemodynamics, metabolic parameters, mitochondrial function, and inflammation during resuscitation from hemorrhagic shock (HS) in mice with pre-traumatic cigarette smoke (CS) exposure with the effects on lung function already published in a previous paper (17).

Neither an attenuation of GR function (GR^dim/dim^) nor CS exposure prior to resuscitation from HS showed an effect on survival rates. However, we found that non-CS exposed GR^dim/dim^ mice needed significantly higher doses of norepinephrine (NE) to keep hemodynamic stability compared to GR^+/+^ mice. Furthermore, lower pH and higher lactate levels as well as an increase in systemic cytokines was present in non-CS GR^dim/dim^ compared to GR^+/+^ mice. Surprisingly, these genotype dependent differences disappeared in CS exposed animals.

Compared to a mouse model based on lipopolysaccharide (LPS)-induced systemic inflammation, we previously reported a significantly increased mortality in GR^dim/dim^ mice compared to GR^+/+^ mice (16). In that LPS model, GR^dim/dim^ mice presented with significant higher need for NE to keep hemodynamic stability, like in the present study the non-CS exposed GR^dim/dim^ mice during resuscitation from HS. However, in the present study there was no effect on survival. Although resuscitation manners (except for colloids) as well as definition for hemodynamic stability (MAP≥55mmHg) were similar in both studies, with regards on survival rates it must be considered that the observation time in the LPS study was 6 h and in the present study 4 hours. If the observation time would have been 6 h in the present study, there may would have been more animals not surviving the whole observation period after HS in the non-CS GR^dim/dim^ group with a probably significant effect on survival rates. Interestingly, CS exposure led to a higher hemodynamic stability in GR^dim/dim^ mice following HS shown by a trend towards a decreased dose of NE in the present study. We can only speculate for possible reasons for this effect of CS exposure on hemodynamics. A possible increased sympathetic activity with reduced demand of additional catecholamine support in CS exposed mice during resuscitation from HS has been reported before (21). This may have been the reason for the absolute lower catecholamine need in CS exposed GR^dim/dim^ mice during resuscitation from HS compared to non-CS exposed animals. An interaction of CS exposure and glucocorticoid receptor (GR) function might also be a possible explanation for the lower NE need in CS exposed animals, because one study reported that maternal tobacco smoke exposure increased serum corticosterone levels in adult male rat offspring (27). Another study reported that CS exposure during breastfeeding in rats showed changes in serum corticosterone level in in obese adult offspring (28). However, with the results in the present study, we can only speculate about possible effects of CS exposure on GR function. This needs further investigation.

In a previous study from our group with a similar murine model of resuscitation from HS, subsequent CS exposure per se had the strongest impact on inflammatory responses, shown by an increased systemic inflammation (21). Furthermore, in a murine model of blunt chest trauma (without HS), pre-traumatic CS exposure also enhanced post-traumatic inflammation (8). In the present study, an increased systemic inflammation could only be seen in part due to the attenuation of GR function in non-CS mice (increased plasma IL-6, IL-12 p40, and Eotaxin in non-CS GR^dim/dim^ compared to non-CS GR^+/+^ mice, **Table 2**), whereas there were no differences in systemic cytokines in CS exposed mice. Although anti-inflammatory effects *via* CS exposure have been reported in a murine model of allergic asthma (29), such protective effects are more unlikely in the present study, because our CS exposure model has, so far, only shown pro-inflammatory effects in our murine trauma models (8, 21). As mentioned above, the short observation time of 4h also might be a reason for the missing inflammatory effect of CS exposure in the present murine trauma model.

With regards to metabolic parameters, in the present study only GR^dim/dim^ mice without a CS exposure presented with a lower pH, a lower base excess (BE), and higher lactate levels compared to GR^+/+^ mice (**Table 1**). The higher lactate levels are in agreement with the study by Vandewalle and colleagues showing an impaired lactate clearance upon LPS-exposure in GR^dim/dim^ mice even in the absence of resuscitation (30). With regards to the lower pH and lower BE in non-CS GR^dim/dim^ mice shown in the present study it must be considered that the lactate levels alone cannot be the reason for this metabolic acidosis. A metabolic acidosis in mice due to other fixed acids than lactic acid has been reported before (11, 24). Furthermore, in the present study, pH, BE, and lactate levels were similar in non-CS exposed GR^+/+^ and CS exposed GR^+/+^ and GR^dim/dim^ mice with a trend towards a slightly lower pH in the latter group, which might have been induced by the higher P_a_CO_2_ values (**Table 1**). Therefore, CS exposure had no effects on pH, BE, and lactate levels on GR^+/+^ mice in the present study. This is in line with previous studies also showing no effect of CS exposure on pH or lactate levels in a more recent mouse model of HS (21).

Hemodynamic instability coincides with an increased need of catecholamines. It is well known that both, endogenously produced as well as externally administered catecholamines exert non-hemodynamic effects and impact metabolism (31). Vital organs such as heart, brain, and kidney may receive energy under stress situations like HS through release of lactate from skeletal muscle as well as by breaking down amino acids, glycogen, and triglycerides to generate glucose, fatty acids, and ketone bodies, all mediated *via* an increased release of endogenous catecholamines (32, 33). Therefore, the increased glucose level in CS exposed GR^dim/dim^ mice in the present study could have been induced *via* increased doses of catecholamines. However, the need of catecholamines to keep hemodynamic stability was higher in non-CS exposed GR^dim/dim^ mice with no effect on glucose levels compared to GR^+/+^ mice in this group. Furthermore, we found no differences in endogenous glucose production or aerobic glucose oxidation between all groups of mice (**Figure 3**). Therefore, the increased glucose concentration in CS exposed GR^dim/dim^ mice does not come from an increased dose of catecholamines or an increase in gluconeogenesis or decrease in glucose oxidation. Moreover, it is most likely an effect of a reduced peripheral uptake of glucose in tissue which utilizes glucose in a non-oxidative way in this group of mice. We can only speculate for possible reasons for this effect. On one hand, in our subsequent studies in LPS-challenged GR^dim/dim^ and GR^+/+^ mice we found no differences for glucose concentrations between GR^dim/dim^ and GR^+/+^ mice (16). On the other hand, in previous studies CS exposure in wildtype mice lead to a trend towards lower glucose levels (21, 24). Therefore, the increased glucose level in CS exposed GR^dim/dim^ mice in the present study are more likely related to both, the combination of CS exposure and attenuation of GR function, which warrants further investigation.

Besides a catecholamine-induced energy production *via* increased carbohydrate, catecholamines also mediate energy production *via* a ketotic effect through an increase in fat and protein oxidation (32, 34). The ketotic impacts of both epinephrine and NE are similar at physiologically low concentrations, whereas the ketotic effect of NE predominates at pathophysiological concentrations, possibly due to an antiketogenic effect resulting from the epinephrine-induced increase of glucose and subsequent insulin concentrations (31), with the latter one may be missing in the present study. With measurements of the rate of appearance of urea in the present study, we were able to determine hepatic metabolic capacity as well as in part kidney function. After CS exposure, GR^dim/dim^ mice produced significantly less urea compared to non-CS GR^dim/dim^ mice and had a trend towards a lower urea production compared to CS exposed GR^+/+^ mice (**Figure 4A**). With no differences between the groups for kidney function measured *via* plasma creatinine concentration, creatinine clearance, and urine output in the present study we can exclude differences in urea excretion *via* the kidney (**Figures 4A, D**). With no differences in rate of appearance for leucine (protein degradation, **Figure 4B**), we can also exclude differences in urea metabolized to amino acids. Therefore, primarily the reduced urea in CS exposed GR^dim/dim^ mice after resuscitation from HS in the present study is most likely a result of a reduced hepatic synthesis. However, this is in contrast with the results of the rate of appearance for glucose, since both, urea and glucose are metabolized in the periportal cells and therefore we should have seen effects on both, rate of appearance in urea and glucose (35).

Last, in CS exposed GR^dim/dim^ mice the rate of appearance for glycerol was higher compared to CS exposed GR^+/+^ mice, which indicates an increase in lipolysis. However, with no differences in plasma corticosterone concentrations in CS exposed GR^dim/dim^ and GR^+/+^ mice in the present study, it is at least less likely that differences in corticosterone concentrations due to an attenuation of GR function might have altered glucose or lipid metabolism in GR^dim/dim^ mice in the present study.

## Conclusion

Cigarette smoke (CS) exposure before hemorrhagic shock (HS) led to an increased hemodynamic stability in mice with an attenuated GR function (GR^dim/dim^), indicated by a trend towards an absolute lower norepinephrine (NE) need. CS exposed GR^dim/dim^ mice also presented with less metabolic changes (pH, base excess, and lactate levels), and showed an increase in lipid oxidation compared to non-CS GR^dim/dim^ mice. If the more stable pH and BE values, the lower lactate levels, or the switch to lipid oxidation to possibly increase the efficiency of energy production to may maintain organ function were the reason or the consequence for the lower NE need in these mice warrants further investigation.

## Data availability statement

The raw data supporting the conclusions of this article will be made available by the authors, without undue reservation.

## Ethics statement

The animal study was reviewed and approved by Regierungspraesidium Tuebingen, Baden-Wuerttemberg, Germany.

## Author contributions

MW, JP, PR, JT, and SV conceived and designed the study. MW, JP, TM, UW, CT, MK, JV, SK, MG, MF, EC, UB, PR, and SV performed the experiments and organ analysis. MW, JP, TM, UW, JV, EC, PR, JT, and SV analyzed the data and interpreted the results. MW, JP, and SV prepared the figures. MW, JP, and SV wrote the manuscript. PR and JT revised the manuscript. All authors contributed to the article and approved the submitted version.

## Funding

Publication costs are funded by the Deutsche Forschungsgemeinschaft (DFG, German Research Foundation)—Project ID INST 40/600-1—Collaborative Research Center (CRC) Projektnummer 251293561 - SFB 1149 to MW and SV. SV received funding from Deutsche Forschungsgemeinschaft (DFG, German Research Foundation), GRK 2203 and DFG VE 994/2-1. PR and JT received funding from the Deutsche Forschungsgemeinschaft (DFG, German Research Foundation)—Collaborative Research Center (CRC) Projektnummer 251293561 - SFB 1149 and DFG Tu 220/13-1 to JT.

## Conflict of interest

Authors CT, MK, and JB are employed by Boehringer Ingelheim Pharma GmbH & Co KG.

The remaining authors declare that the research was conducted in the absence of any commercial or financial relationships that could be construed as a potential conflict of interest.

## Publisher’s note

All claims expressed in this article are solely those of the authors and do not necessarily represent those of their affiliated organizations, or those of the publisher, the editors and the reviewers. Any product that may be evaluated in this article, or claim that may be made by its manufacturer, is not guaranteed or endorsed by the publisher.

## References

[1] Angele MK Schneider CP Chaudry IH. Bench-to-bedside review: Latest results in hemorrhagic shock. Crit Care (2008) 12(4):218. doi: 10.1186/cc6919 18638356 PMC2575549

[2] Denk S Weckbach S Eisele P Braun CK Wiegner R Ohmann JJ. Role of hemorrhagic shock in experimental polytrauma. Shock (2017) 49(2):154–63. doi: 10.1097/SHK.0000000000000925 28614141

[3] Eltzschig HK Carmeliet P. Hypoxia and inflammation. N Engl J Med (2011) 364(7):656–65. doi: 10.1056/NEJMra0910283 PMC3930928 21323543

[4] Douzinas EE Livaditi O Tasoulis M-K Prigouris P Bakos D Goutas N. Nitrosative and oxidative stresses contribute to post-ischemic liver injury following severe hemorrhagic shock: The role of hypoxemic resuscitation. PLoS One (2012) 7(3):e32968. doi: 10.1371/journal.pone.0032968 22403729 PMC3293918

[5] Merz T Denoix N Huber-Lang M Singer M Radermacher P McCook O. Microcirculation vs. mitochondria-what to target? Front Med (Lausanne) (2020) 7:416. doi: 10.3389/fmed.2020.00416 32903633 PMC7438707

[6] Barth E Albuszies G Baumgart K Matejovic M Wachter U Vogt J. Glucose metabolism and catecholamines. Crit Care Med (2007) 35(9 Suppl):S508–18. doi: 10.1097/01.CCM.0000278047.06965.20 17713401

[7] Vanhorebeek I Gunst J Ellger B Boussemaere M Lerut E Debaveye Y. Hyperglycemic kidney damage in an animal model of prolonged critical illness. Kidney Int (2009) 76(5):512–20. doi: 10.1038/ki.2009.217 19536085

[8] Wagner K Gröger M McCook O Scheuerle A Asfar P Stahl B. Blunt chest trauma in mice after cigarette smoke-exposure: Effects of mechanical ventilation with 100% O2. PloS One (2015) 10(7):e0132810. doi: 10.1371/journal.pone.0132810 26225825 PMC4520521

[9] Geldmacher H Biller H Herbst A Urbanski K Allison M Buist AS. Die prävalenz der chronisch obstruktiven lungenerkrankung (COPD) in deutschland. Ergebnisse der BOLD-Studie Dtsch Med Wochenschr (2008) 133(50):2609–14. doi: 10.1055/s-0028-1105858 19052996

[10] Sinden NJ Stockley RA. Systemic inflammation and comorbidity in COPD: a result of 'overspill' of inflammatory mediators from the lungs? Review of the evidence. Thorax (2010) 65(10):930–6. doi: 10.1136/thx.2009.130260 20627907

[11] Hartmann C Hafner S Scheuerle A Möller P Huber-Lang M Jung B. The role of cystathionine-γ-Lyase in blunt chest trauma in cigarette smoke exposed mice. Shock (2017) 47(4):491–9. doi: 10.1097/SHK.0000000000000746 27685807

[12] Minei JP Cuschieri J Sperry J Moore EE West MA Harbrecht BG. The changing pattern and implications of multiple organ failure after blunt injury with hemorrhagic shock. Crit Care Med (2012) 40(4):1129–35. doi: 10.1097/CCM.0b013e3182376e9f PMC3343366 22020243

[13] Vettorazzi S Nalbantoglu D Gebhardt JCM Tuckermann J. A guide to changing paradigms of glucocorticoid receptor function-a model system for genome regulation and physiology. FEBS J (2021). doi: 10.1111/febs.16100 34213830

[14] Lim H-W Uhlenhaut NH Rauch A Weiner J Hübner S Hübner N. Genomic redistribution of GR monomers and dimers mediates transcriptional response to exogenous glucocorticoid *in vivo* . Genome Res (2015) 25(6):836–44. doi: 10.1101/gr.188581.114 PMC4448680 25957148

[15] Vettorazzi S Bode C Dejager L Frappart L Shelest E Klaßen C. Glucocorticoids limit acute lung inflammation in concert with inflammatory stimuli by induction of SphK1. Nat Commun (2015) 6:7796. doi: 10.1038/ncomms8796 26183376 PMC4518295

[16] Wepler M Preuss JM Merz T Hartmann C Wachter U McCook O. Impaired glucocorticoid receptor dimerization aggravates LPS-induced circulatory and pulmonary dysfunction. Front Immunol (2019) 10:3152. doi: 10.3389/fimmu.2019.03152 32038649 PMC6990631

[17] Preuss JM Burret U Gröger M Kress S Scheuerle A Möller P. Impaired glucocorticoid receptor signaling aggravates lung injury after hemorrhagic shock. Cells (2021) 11(1):112. doi: 10.3390/cells11010112 35011674 PMC8750862

[18] Langgartner D Wachter U Hartmann C Gröger M Vogt J Merz T. Effects of psychosocial stress on subsequent hemorrhagic shock and resuscitation in Male mice. Shock (2019) 51(6):725–30. doi: 10.1097/SHK.0000000000001204 29889818

[19] Gröger M Wepler M Wachter U Merz T McCook O Kress S. The effects of genetic 3-mercaptopyruvate sulfurtransferase deficiency in murine traumatic-hemorrhagic shock. Shock (2018) 51(4):472–478. doi: 10.1097/SHK.0000000000001165 PMC6192867 29668565

[20] Gröger M Hogg M Abdelsalam E Kress S Hoffmann A Stahl B. Effects of sodium thiosulfate during resuscitation from trauma-and-Hemorrhage in cystathionine gamma lyase (CSE) knockout mice. Shock (2022) 57(1):131–9. doi: 10.1097/SHK.0000000000001828 34172609

[21] Hartmann C Gröger M Noirhomme J-P Scheuerle A Möller P Wachter U. In-depth characterization of the effects of cigarette smoke exposure on the acute trauma response and hemorrhage in mice. Shock (2018) 51(1):68–77. doi: 10.1097/SHK.0000000000001115 29424792

[22] Reichardt HM Kaestner KH Tuckermann J Kretz O Wessely O Bock R. DNA Binding of the glucocorticoid receptor is not essential for survival. Cell (1998) 93(4):531–41. doi: 10.1016/s0092-8674(00)81183-6 9604929

[23] Merz T Kress S Gröger M Radermacher P McCook O. Mouse intensive care unit (MICU). Methods Mol Biol (2021) 2321:121–35. doi: 10.1007/978-1-0716-1488-4_11 34048012

[24] Hafner S Wagner K Weber S Gröger M Wepler M McCook O. Role of the purinergic receptor P2XR4 after blunt chest trauma in cigarette smoke-exposed mice. Shock (2017) 47(2):193–9. doi: 10.1097/SHK.0000000000000726 27559703

[25] Wollin L Pieper MP. Tiotropium bromide exerts anti-inflammatory activity in a cigarette smoke mouse model of COPD. Pulm Pharmacol Ther (2010) 23(4):345–54. doi: 10.1016/j.pupt.2010.03.008 20362689

[26] Vogt JA Wachter U Wagner K Calzia E Gröger M Weber S. Effects of glycemic control on glucose utilization and mitochondrial respiration during resuscitated murine septic shock. Intensive Care Med Exp (2014) 2(1):19. doi: 10.1186/2197-425X-2-19 26266919 PMC4678133

[27] Zinkhan EK Lang BY Yu B Wang Y Jiang C Fitzhugh M. Maternal tobacco smoke increased visceral adiposity and serum corticosterone levels in adult male rat offspring. Pediatr Res (2014) 76(1):17–23. doi: 10.1038/pr.2014.58 24727947

[28] Novaes Soares P Silva Tavares Rodrigues V Cherem Peixoto T Calvino C Aparecida Miranda R Pereira Lopes B. Cigarette smoke during breastfeeding in rats changes glucocorticoid and vitamin d status in obese adult offspring. Int J Mol Sci (2018) 19(10):3084. doi: 10.3390/ijms19103084 PMC6213898 30304827

[29] Tilp C Bucher H Haas H Duechs MJ Wex E Erb KJ. Effects of conventional tobacco smoke and nicotine-free cigarette smoke on airway inflammation, airway remodelling and lung function in a triple allergen model of severe asthma. Clin Exp Allergy (2016) 46(7):957–72. doi: 10.1111/cea.12665 26502779

[30] Vandewalle J Timmermans S Paakinaho V Vancraeynest L Dewyse L Vanderhaeghen T. Combined glucocorticoid resistance and hyperlactatemia contributes to lethal shock in sepsis. Cell Metab (2021) 33(9):1763–76.e5. doi: 10.1016/j.cmet.2021.07.002 34302744

[31] Hartmann C Radermacher P Wepler M Nußbaum B. Non-hemodynamic effects of catecholamines. Shock (2017) 48(4):390–400. doi: 10.1097/SHK.0000000000000879 28915214

[32] Gjedsted J Buhl M Nielsen S Schmitz O Vestergaard ET Tønnesen E. Effects of adrenaline on lactate, glucose, lipid and protein metabolism in the placebo controlled bilaterally perfused human leg. Acta Physiol (Oxf) (2011) 202(4):641–8. doi: 10.1111/j.1748-1716.2011.02316.x 21624100

[33] Steinberg D Nestel PJ Buskirk ER Thompson RH. Calorigenic effect of norepinephrine correlated with plasma free fatty acid turnover and oxidation. J Clin Invest (1964) 43:167–76. doi: 10.1172/JCI104901 PMC289510 14162525

[34] Bearn AG Billing B Sherlock S. The effect of adrenaline and noradrenaline on hepatic blood flow and splanchnic carbohydrate metabolism in man. J Physiol (1951) 115(4):430–41. doi: 10.1113/jphysiol.1951.sp004679 PMC1392027 14898520

[35] Li L Zhang P Bao Z Wang T Liu S Huang F. PGC-1α promotes ureagenesis in mouse periportal hepatocytes through SIRT3 and SIRT5 in response to glucagon. Sci Rep (2016) 6:24156. doi: 10.1038/srep24156 27052737 PMC4823758

