# Supplementary material for: Cigarette smoke exposure reduces hemorrhagic shock induced circulatory dysfunction in mice with attenuated glucocorticoid receptor function
Source: Front Immunol. 2022 Sep 12;13:980707. doi: 10.3389/fimmu.2022.980707 (PMC9510589; doi:10.3389/fimmu.2022.980707)
Supplement: Supplementary file 1 [file DataSheet_1.docx]

**Supplemental Material**

*Analysis of metabolic pathways and kidney function*

^13^C_6_-glucose, 6,6-^2^H_2_-glucose, ^15^N_2_-urea and ^2^H_5_-glycerol were obtained from Campro Scientific (Berlin, Germany), 5,5,5-^2^H_3_-leucine from CIL (Tewksbury, MA, USA) and ^2^H_3_-creatinine from CDN isotopes (Pointe-Claire, Quebec, Canada). Derivatization compounds were purchased from ABCR (Karlsruhe, Germany), methyl-urea and solvents in p.a. quality from Sigma-Aldrich (Munich, Germany) and cation-exchange extraction cartridges from Phenomenex (Aschaffenburg, Germany). Rates of appearances (endogenous production rates) of glucose, urea, glycerol and leucine were measured to monitor gluconeogenesis, nitrogen turnover, lipolysis and protein breakdown. Glucose oxidation rate was assessed from the expiratory ^13^CO_2_ release, determined as product of total expiratory CO_2_ concentration and ^13^CO_2_ enrichment. Additionally, plasma levels of glucose and urea were determined. Creatinine clearance as parameter for kidney function was calculated form excreted creatinine and creatinine plasma concentrations. After sample thawing, samples were spiked with internal standards (6,6-^2^H_2_-glucose, ^2^H_3_-creatinine and N-methyl-urea for quantification of glucose, creatinine and urea), deproteinized and purified by cation-exchange solid-phase-extraction (separation of creatinine from interfering creatine and creatinephosphate). Next steps were derivatization and GC/MS analysis.

Glucose and glycerol were derivatized with N-Methyl-bis (trifluoroacetamide) (MBTFA) to the corresponding trifluoroacetates. Urea and leucine were converted with N-(tert-butyldimethylsilyl)-N-methyltrifluoroacetamide (MTBSTFA) to tert.-butyl-dimethylsilyl derivatives. Creatinine was analyzed as trimethylsilyl derivative after reaction with N,O-bis(trimethylsilyl)trifluoroacetamide (BSTFA). Acetonitrile was used as solvent for all derivatization reactions. GC/MS determinations were performed with an Agilent 5890/5970 GC/MS system housing a MN Optima-5-MS capillary column (12mx0.2mm, 0.35µm film thickness; Macherey-Nagel, Düren, Germany). The MS was operated in electron impact ionization in the selected ion monitoring mode.

Plasma concentrations of glucose, urea and creatinine, as well as the urinary creatinine concentration were determined with a six-point calibration curve. Peak area ratios of the endogenous compound vs. the added internal standard were plotted against amount ratios to generate calibration curves.

*Calculations*

Rates of appearances (endogenous production of gluconeogenesis, glycerol, urea and leucine production) were calculated according to equation 1 from isotope infusion rates and the measured ratio of labeled vs unlabeled compounds in plasma.

Ra = Inf / TTR (Eq.1)

Ra: rate of appearance

Inf: infusion rate of stable isotope labelled compound

TTR: tracer (isotope labeled compound) to tracee (endogenous compound) ratio

Glucose oxidation rates were calculated from ^13^C_6_-glucose infusion rates, total CO_2_ release and expiratory ^13^CO_2_ enrichments according to equation 2-4.

OR = V_13_co_2_ / Inf_13C_ (Eq.2)

OR = (TTR_13CO2_ / (TTR_13CO2_ +1)) * C_CO2_ *RMV / Inf_13C_ (Eq.3)

OR = (TTR_13CO2_ / (TTR_13CO2_ +1)) * C_CO2_ *RMV / (Inf_13C6-Gluc_ * 6) (Eq.4)

OR: glucose oxidation rate

V_13_co_2_: total expiratory CO_2_ release

Inf_13C_: Infusion rate of ^13^C

TTR_13CO2_: tracer to tracee ratio of ^13^CO_2_ in expired air

C_CO2_: total concentration of CO_2_ in expired air

RMV: respiratory minute volume

Inf_13C6-Gluc_ Infusion rate of ^13^C_6_-glucose

Creatinine clearance was calculated from plasma and urinary creatinine concentrations, urine volume and the urine sampling period:

CC = (Crea_U_ * Vol_U_) / (Crea_Pl_ * t)

CC: creatinine clearance

Crea_U_: urinary creatinine concentration

Vol_U_: volume of sampled urine

Crea_Pl_: plasma creatinine concentration

t: urine sampling time
